# Co-Production of Dimethyl Carbonate, Dimethoxymethane and Dimethyl Ether from Methanol: Process Design and Exergy Analysis

**DOI:** 10.3390/e24101438

**Published:** 2022-10-09

**Authors:** Shuxing Zhang, Xiaoshu Ding, Helen Shang, Yucong Song, Yanji Wang

**Affiliations:** 1 School of Chemical Engineering and Technology, Hebei University of Technology, Tianjin 300130, China; 2 Bharti School of Engineering, Laurentian University, Sudbury, ON P3E 2C6, Canada

**Keywords:** dimethyl carbonate, Aspen Plus, co-production process, exergy analysis

## Abstract

Dimethyl carbonate is an important green chemical that has been widely used in the chemical industry. In the production of dimethyl carbonate, methanol oxidative carbonylation has been studied, but the conversion ratio of dimethyl carbonate using this method is too low, and the subsequent separation requires a large amount of energy due to methanol and dimethyl carbonate being azeotrope. In this paper, the strategy of “reaction instead of separation” is proposed. Based on this strategy, a novel process is developed to combine the production of DMC with that of dimethoxymethane (DMM) and dimethyl ether (DME). The co-production process was simulated using Aspen Plus software, and the product purity was up to 99.9%. The exergy analysis of the co-production process and the existing process was carried out. The exergy destruction and exergy efficiency were compared with those of the existing production processes. The results show that the exergy destruction of the co-production process is about 276% less than that of the single-production processes, and the exergy efficiencies in the developed co-production process are significantly improved. The utility loads of the co-production process are significantly lower than that of the single-production process. The developed co-production process increases the methanol conversion ratio to 95%, with a reduced energy requirement. It is proved that the developed co-production process can provide an advantageous option over the existing processes with improved energy efficiency and material savings. The strategy of “reaction instead of separation” is feasible. A new strategy is proposed for azeotrope separation.

## 1. Introduction

Dimethyl carbonate (DMC) is an important green chemical [1] and can replace poisonous phosgene and dimethyl sulfate as the carbonylation or methylation agent due to the various functional groups in its molecule, such as methyl, methoxy, and carbonyl groups [2]. DMC can be used as a gasoline additive to significantly improve the combustion performance and antiknock performance of gasoline by adding 6% of it in gasoline [3]. It can also be used as a diesel additive to reduce the soot emission of diesel engines by adding 4% of it to diesel [4].

DMC can be synthesized by various methods, including those using phosgene, transesterification, alcoholysis of urea, methanol-CO_2_, liquid phase oxidative carbonylation of methanol, and the vapor phase oxidative carbonylation of methanol. Among these methods, the one using vapor phase oxidative carbonylation of methanol has advantages over the others due to its low cost, easy access to feed materials, and simple and environmentally friendly production procedure. Extensive research has focused on DMC production by the vapor phase oxidative carbonylation of methanol, and catalysts play a crucial role in DMC production [5,6,7,8,9,10]. Different catalysts and their performances in DMC productions are listed in Table 1. As shown in Table 1, the methanol conversion ratio is low in DMC production, and thus, a large number of feed materials need to be recycled. Under the various reaction conditions, the highest DMC yield ratio is about 10%, i.e., only 10% of methanol reacts to form DMC [5]. The produced DMC needs to be separated from the unreacted methanol. Because methanol and DMC form an azeotrope, pressure swing distillation or extractive distillation are usually used for separation [11,12]. The former has high energy consumption, while the latter requires the introduction of an extractant. Due to the limitations of using gas-phase oxidative carbonylation, the development of a novel DMC production approach with improved methanol conversion ratio and energy efficiency is desirable.

In this paper, the strategy of “reaction instead of separation” is proposed. Based on this strategy, a novel process is developed to combine the production of DMC with that of dimethoxymethane (DMM) and dimethyl ether (DME). In this method, three reactions occur sequentially to generate DMC, DMM, and DME, respectively. DMC is first generated from carbon monoxide, methanol, and oxygen. Due to the low methanol conversion in the DMC reaction, a large amount of methanol is not reacted and therefore enters the second reactor. The reaction occurs in the second reactor to produce DMM, and the remaining carbon monoxide is also consumed. The unreacted methanol then enters the third reactor, where more methanol is converted to DME. Having three reactions in a series significantly increases the methanol conversion ratio and reduces the cost resulting from recycling a large amount of unreacted methanol. Three products—DMC, DMM, and DME—are produced from the procedure.

The products from the proposed process have wide applications. Dimethoxymethane (DMM) has been used in rubber, paints, inks, and other industrial processes. DMM is commonly produced by catalytic oxidation of methanol [13,14,15], as it is more economical and environmentally friendly than the aldol condensation method. However, the catalytic oxidation method has difficulties in separating dimethoxymethane from methanol [16,17]. Pressure swing distillation is usually used for the separation of DMM and methanol. Under various reaction conditions, the catalyst can achieve a DMM yield of 57.6% [18].

Dimethyl ether (DME) can be used as a source of energy due to its versatility and low price. The gas-phase dehydration of methanol is often used to produce DME. The catalyst prepared by Yu Sang can reach a DME yield of 88.7% [19]. In the proposed strategy, DMC, DMM, and DME are produced sequentially using three reactions, and the products are then separated using distillation columns. In this co-production, high yields can be achieved for all three reactions at low temperatures [5,8,19], and thus, the reactions do not require a large amount of energy. The high energy efficiency of this method means that it is very attractive in large-scale productions. In this paper, the co-production process is simulated using Aspen Plus and compared with the existing single-production processes. Exergy destruction and exergy efficiencies are analyzed for the process. Exergy analysis uses mass and energy conservations and the second law of thermodynamics, and it can be used to estimate the internal energy losses of a system and identify the losses as a result of irreversibility [20]. Exergy analysis indicates that the proposed process possesses high energy efficiency.

## 2. Process Description

**Figure 1:**
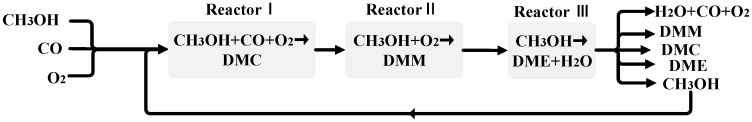
Schematic of co-production of DMC, DMM and DME (3DM) from methanol.

The process is designed such that DMC, DMM, and DME (3DM) are produced sequentially, as shown in Figure 1.

The first step is the gas-phase oxidative carbonylation of methanol to produce DMC. The reaction in Reactor I is as follows:

(1) $$4{\mathrm{CH}}_{3}\mathrm{OH}+2\mathrm{CO}+O_{2}\to 2{\left({\mathrm{CH}}_{3}O\right)}_{2}\mathrm{CO}+2H_{2}O\;\Delta H_{R}=-149.75\;\mathrm{kJ}/\mathrm{mol}$$

The reaction occurs with PdCl_2_-CuCl_2_-KOAc/AC as a catalyst [5]. At 423.15 K and 0.3 MPa, the conversion of methanol was 12%, and the selectivity of dimethyl carbonate to methanol was 80%. After the reaction, the reactants and products enter the second reactor. In Reactor II, DMM is synthesized by the one-step oxidation of methanol, as follows:

(2) $$6{\mathrm{CH}}_{3}\mathrm{OH}+O_{2}\to 2{\mathrm{CH}}_{2}{\left({\mathrm{OCH}}_{3}\right)}_{2}+4H_{2}O\;\Delta H_{R}=-76.34\;\mathrm{kJ}/\mathrm{mol}$$

The reaction occurs with VO_x_/TiO_2_ nanotube-SO_4_^2−^ as a catalyst, as prepared by Shen Yi [18]. At 403 K and the atmospheric pressure, DMM was obtained with methanol conversion of 64% and DMM selectivity up to 90%. After the reaction, there is still a large amount of unreacted methanol in the stream. The unreacted methanol and other components go to the third reactor for further reaction.

In the third reactor, methanol is dehydrated to form DME. The reaction is as follows:

(3) $$2{\mathrm{CH}}_{3}\mathrm{OH}\to {\mathrm{CH}}_{3}{\mathrm{OCH}}_{3}+H_{2}O\;\Delta H_{R}=12.02\;\mathrm{kJ}/\mathrm{mol}$$

The reaction occurs with H-ZSM-5/MCM-41 composite molecular sieve as a catalyst. Under atmospheric pressure and 493.15 K, the methanol conversion ratio reaches 88.7%, and the dimethyl ether selectivity is 100% [19].

After the three reactions, the outlet stream contains methanol, carbon monoxide, water, oxygen, DMM, DME, and DMC. Carbon monoxide and oxygen are in the gas phase, and the other materials are liquid. The gas–liquid separator divides the gas from the liquid stream. After separating DME using distillation, there are water, DMM, DME, and methanol in the liquid stream. Distillation is then used to separate the liquid into two streams, at the top of the column are DMM and methanol, and at the bottom of the column are DMC and water. Pressure swing distillations are then used to obtain DMM and DMC from their respective mixtures.

## 3. Process Model Development

In this section, a model is built for the 3DM co-production using Aspen Plus, and a simulation is carried out. In the model, the WILSON property is adopted. For the reactors, the RStoic module is selected using the known temperature, pressure, and yield of the reactions based on the experimental data [5,18,19]. Assuming that the gas and liquid can be completely separated, the Sep module is selected for the gas–liquid separator. The RadFrac module is selected for the distillation columns. The Heater module is selected for the preheater. The Mixer module is selected for the mixer. The Pump module is selected for the booster pump. The model is developed based on the following assumptions:

(1) The system is in a steady state [21].
(2) The reference temperature (T_0_) and reference pressure (P_0_) are constant.
(3) The Sep module, the Heater module, the Mixer module, and the Pump module are chosen as adiabatic. The RStoic module and the RadFrac module are chosen as non-adiabatic.
(4) The exergy terms for kinetic and gravitational potential energy are negligible [22,23].
(5) The chemical exergy terms for the process units are constant.
(6) Side reactions can be neglected as the selectivity of all three reactions is above 80%.
(7) All streams are ideal mixtures.

**Figure 2:**
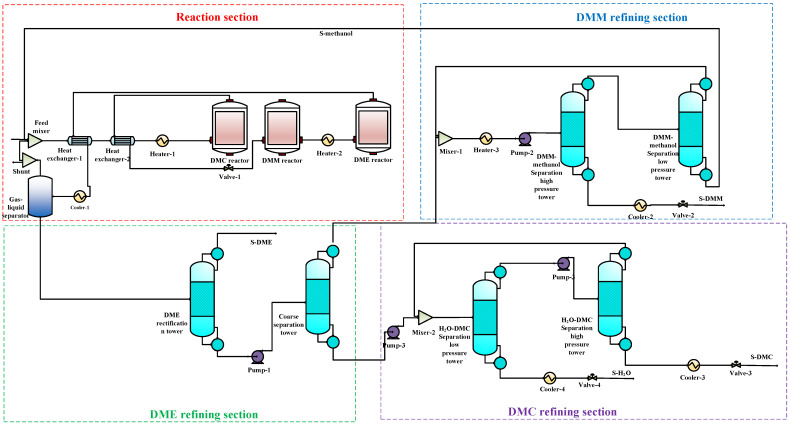
The 3DM co-production process flow chart.

The 3DM co-production process(all the data was shown in the Supplementary Materials) includes two parts: the reaction system and the separation system. In this paper, Aspen Plus (V8.4, AspenTech, Bedford, USA) is used to establish the 3DM co-production process, as shown in Figure 2. The reaction system comprises a mixer, a preheater, and three reactors. The feed materials are mixed in the mixer, preheated in the preheater, and then sequentially enter the DMC reactor, DMM reactor, and DME reactor. The reaction conditions are set to be consistent with the experimental data [5,18,19]. The separation system mainly includes a gas–liquid separator, a DME rectification column, a crude separation column, a DMM-methanol separation high-pressure column, a DMM-methanol separation low-pressure column, an H_2_O-DMC separation high-pressure column, and an H_2_O-DMC separation low-pressure column. The material out of the reaction system contains DMC, DMM, DME, and unreacted feed materials (oxygen, carbon monoxide, and methanol). The oxygen and carbon monoxide are in the gas phase and can thus be separated from other components using the gas–liquid separator. A small part of the unreacted gas is released, and most of it enters the Feed Mixer to participate in the reaction again. Since DME has a low boiling point and does not form an azeotrope with other components, it can be easily separated by rectification. Out of the DME rectification column, the DME product and a mixture of H_2_O, DMM, methanol, and DMC are obtained. The mixture of H_2_O, DMM, methanol, and DMC enters the crude separation column for separation. Methanol and DMM come out of the top of the column, and H_2_O and DMC leave the column from the bottom. The separations of DMM from methanol and DMC from H_2_O are challenging as DMM and methanol form an azeotrope, as well as H_2_O and DMC. For separations of the azeotropes, pressure swing distillation or extractive distillation are commonly used. The pressure swing distillation is easy to control and does not introduce any new agents, and thus, avoids the need for additional subsequent separation. Pressure swing distillation is, therefore, chosen to separate the two pairs of azeotropes, methanol-DMM and H_2_O-DMC, respectively. The separated methanol is recycled to the feed-materials mixer.

Based on the model in Figure 2, the material balance is calculated for each stream in the process using Aspen Plus V8.4, and the result is shown in Table S1. It can be seen that, after the process is optimized, the concentrations of the products and water are all more than 99.9%. From this table, the methanol conversion ratio of the 3DM co-production process is calculated, and it reaches 95%. This table will be used to calculate the exergy of the streams in the next section.

**Figure 3:**
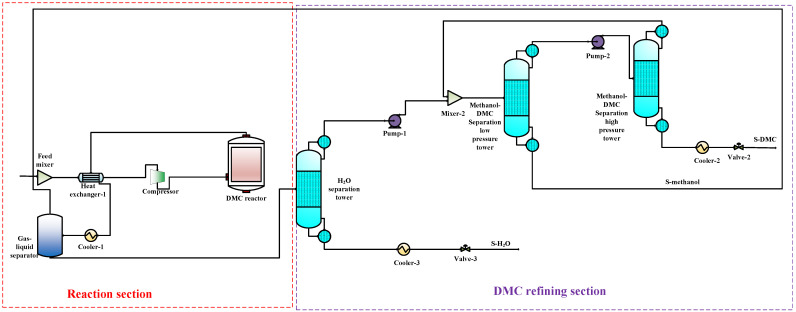
The DMC single-production flow chart.

**Figure 4:**
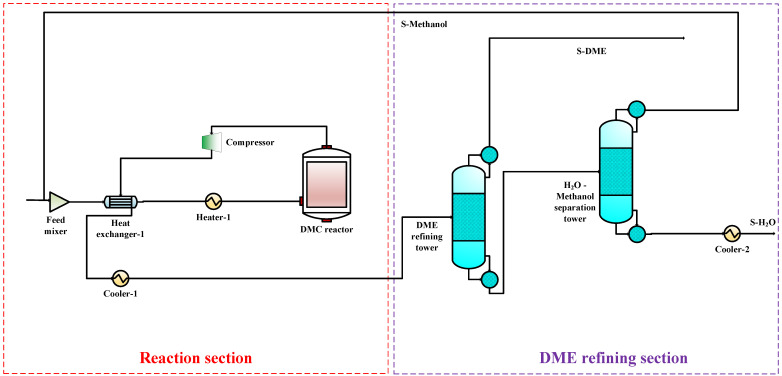
The DME single-production flow chart.

**Figure 5:**
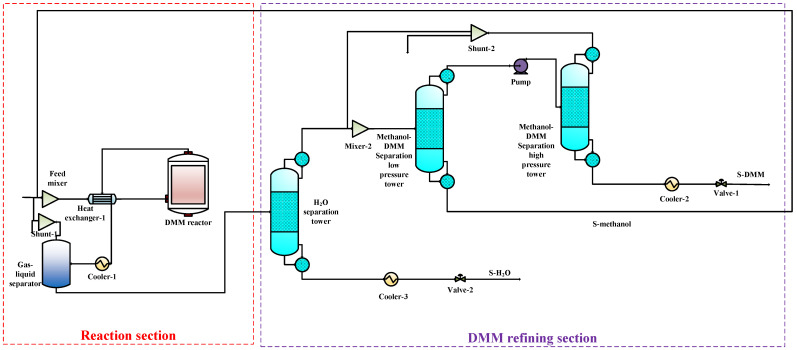
The DMM single-production flow chart.

To compare the performance of the proposed co-production process with that of the DMC, DMM, and DME single-productions, the DMC, DMM, and DME single-production processes are also simulated under the same reaction conditions. The flow charts for DMC, DMM, and DME productions are shown in Figure 3, Figure 4 and Figure 5, respectively. In DMC production, after the reaction generating DMC, H_2_O is separated from the mixture of DMC and methanol using basic distillation. The methanol and DMC are then separated using pressure-swing distillation. The production of DMM is similar to that of DMC. After the reaction producing DMM, H_2_O is separated by rectification, and DMM and methanol are then separated using pressure-swing distillation. For the DME production, after the reaction producing DME, DME is separated from other components by rectification, and H_2_O and methanol are then separated using basic distillation.

## 4. Exergy Analysis

As energy conservation alone is inadequate for depicting some important aspects of energy utilization [22], exergy analysis is performed herein to investigate the 3DM co-production process. The exergy of a system is a measure of the quality of the energy it contains and of its distance from the environment. It is defined as the minimum work necessary to produce a substance in a specific state from the species present in the environment by means of reversible processes in which heat and mass are interchanged only with the surroundings [24]. Exergy is a useful quantity that stems from the Second Law of Thermodynamics [25]. The exergy rate of a stream B can be quantified as follows:

(4) $$B=N(ex^{\mathrm{ch}}+ex^{\mathrm{ph}}+ex^{\mathrm{ki}}+ex^{\mathrm{po}})$$

where $\;ex^{ch}$, $ex^{ph},\;ex^{ki}\;$, and $ex^{po}\;$ are the specific molar chemical exergy, specific molar physical exergy, specific molar kinetic exergy, and specific molar potential exergy, respectively, and N is the molar flow rate. Compared to $ex^{ch}$ and $ex^{ph}$, the values of $ex^{ki}$ and $ex^{po}$ are very small and thus negligible [22,23]. Therefore, only physical exergy and chemical exergy are retained. The specific molar physical exergy ($ex^{ph}$) of a stream can be calculated as follows [26]:

(5) $$ex^{\mathrm{ph}}=H-H_{0}-T_{0}(S-S_{0})$$

where H and  S denote the enthalpy and entropy at the specified pressure and temperature, respectively, $H_{0}\;$ and $S_{0}$ represent the enthalpy and entropy at the Standard Ambient Temperature and Pressure (P_0_ = 0.1 MPa, T_0_ = 298.15 K). The values of H,  S, $\;H_{0}\;$, and $S_{0}$ are available from Aspen plus. The specific molar chemical exergy can be written as [26]:

(6) $$ex^{\mathrm{ch}}=\sum y_{i}Ex_{i}^{o}+RT_{0}\sum y_{i}lny_{i}$$

where $y_{i}\;$ is the mole fraction of component  i in the stream.$\;Ex_{i}^{o}$ is the standard chemical exergy of component i. The standard chemical exergy of the components for the 3DM production is listed in Table 2.

The standard chemical exergy of component i is calculated using the following formula [24,27]:

(7) $$Ex_{i}^{o}=\Delta G_{f}^{0}+\sum_{el}n_{e}{}_{l}Ex_{{}_{\mathrm{qel}}}^{0}$$

where, $∆G_{f}^{0}$ is Gibbs free energy variation in the direction of a given chemical reaction of component $i,\;n_{el}$ is the number of moles of an element $,\;\;Ex_{qel}^{0}$ is the standard chemical exergy of an element.

For an operating unit, the general mass balance is given as [24]:

(8) $$\sum_{}^{}{\overset{•}{m}}_{\mathrm{in}}=\sum_{}^{}{\overset{•}{m}}_{\mathrm{out}}$$

where,$\;{\dot{m}}_{in}$ and ${\dot{m}}_{out}$ are the mass flow rate of inlet and outlet stream, respectively.

$\;B_{d}$ is the internal exergy destruction. The energy and exergy balances for the operating units in the proposed 3DM process are described in Table 3. In Table 3, $\;B_{in}$ and $B_{out}$ are the exergy of the inlet and outlet stream, respectively.${\dot{\;m}}_{in}$ and ${\dot{m}}_{out}$ are mass flow rate of inlet and outbound stream, respectively. *Q_r_* is the high-grade heat sources and *Q_c_* is the low-grade heat sources. *T_r_* is the temperature of the high-grade heat sources and *T_c_* is the temperature of the low-grade heat sources, assuming that the temperature of the cold and hot utilities is a constant.$\;B_{u}\;$ is the exergy rate of the utilities of the process, and it can be written as [28,29]:

(9) $$B_{u}=\left(1-\frac{T_{0}}{T_{r}}\right)Q_{r}-\left(1-\frac{T_{0}}{T_{c}}\right)Q_{c}$$

The exergy associated with work, $Ex^{W},$ can be written as [30]:

(10) $$B_{w}=\overset{•}{W}$$

where $\dot{W}$ is the work rate .

The exergy utilization in the process of percentages φ can defined as [31]

(11) $$\varphi =\frac{\sum B_{p}}{\sum B_{in}+B_{u}}$$

A larger value for exergy efficiency indicates that more exergy enters the product and that the process is more efficient.

## 5. Results and Discussion

Based on the model described in the last section, the exergy of the streams for the 3DM co-production process is calculated and shown in Table S2. From the table, it can be seen that chemical exergy is related to chemical reactions, separation, and mixing. In the absence of a chemical reaction, separation, and mixing, chemical exergy is unchanged. Only the physical exergy changes when there are only heating and cooling processes. Physical exergy is related to the temperature and pressure of the streams and is small compared with chemical exergy.

**Figure 6:**
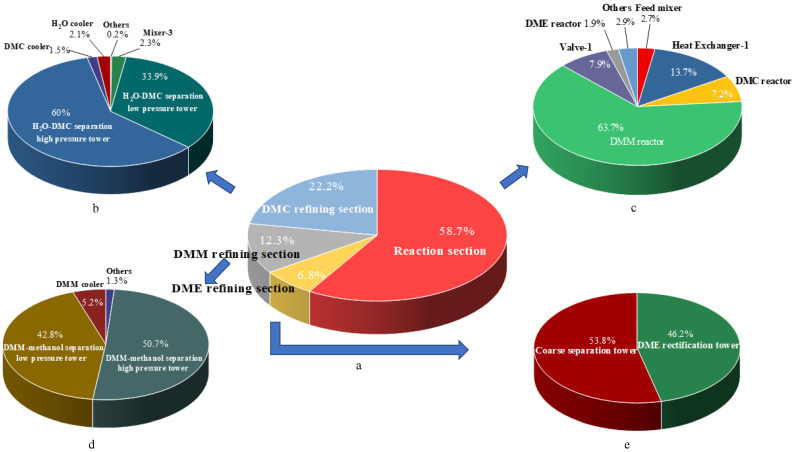
(**a**). The percentage of the internal exergy destruction of each section in 3DM co-production process (**b**). The percentage of the internal exergy destruction of each unit in the DMC refining section (**c**). The percentage of the internal exergy destruction of each unit in the reaction section (**d**). The percentage of the internal exergy destruction of each unit in the DMM refining section (**e**). The percentage of the internal exergy destruction of each unit in the DME refining section.

The 3DM co-production process is divided into four sections, namely the reaction section, the DMM refining section, the DME refining section, and the DMC refining section. The percentages of the internal exergy destruction of the four sections in the 3DM co-production process are shown in Figure 6a. It can be seen from the figure that the sequence of internal exergy losses is reaction section > DMC refining section > DMM refining section > DME refining section. The chemical exergy destruction caused by the chemical reaction is much greater than the physical exergy destruction, so the internal exergy destruction of the reaction section is the largest. DME and other substances do not form azeotropes, so the internal exergy destruction of the DME refining section is minimal. Then, the internal exergy destruction of each section was analyzed separately, as shown in Figure 6b–e. In the reaction section, the internal exergy destruction of the DMM reactor is the largest because the chemical exergy destruction caused by the methanol synthesis DMM reaction is the largest of the other two reactions. In the refining section of DMC, DMM, and DME, the internal exergy destruction of the distillation column accounts for more than 90%.

The internal exergy destruction per unit of product was calculated, as shown in Table 4. It can be seen from Table 4 that the internal exergy destruction for the DME single-production process is smaller than that of the other two single-production processes. This is due to its high methanol conversion ratio and the absence of azeotrope between the product and feed material. For the DMM single-production process, although it involves the separation of azeotropes, the methanol conversion ratio is high, and thus the internal exergy destruction is still smaller than that of the DMC single-production process. In the 3DM co-production process, the products are composed of 12.73% DMC, 43.14% DMM, and 44.13% DME. To produce products with the same composition as the 3DM co-production process, the internal exergy destruction using the single-production processes was calculated to be 1220.676 kJ/mol. The internal exergy destruction for the 3DM co-production saves 896.533 kJ/mol less than that using the single-production processes, accounting for 276% of the internal exergy destruction. This indicates that, by combining the DMM and DME production together with DMC production, the internal exergy destruction is significantly reduced. Based on the definition in the last section, the exergy efficiencies of the 3DM co-production process and the single-production process were calculated and are shown in Table 5. From Table 5, the exergy efficiencies of the DME single-production process are larger than that of DMM single-production process. This is because, in DME production, the feed material and the product do not form an azeotrope, and the methanol conversion ratio is high. Although the single-production DMM process involves the separation of an azeotrope, the reaction yield of the DMM single-production process is larger than that of the DMC single-production process, so the exergy efficiencies  φ  of the DMM single-production process is also higher than that of the DMC single-production process. By combining the DMM, DME, and DMC productions, the proposed 3DM co-production process displays significantly improved the exergy efficiencies  φ. This shows that co-production is advantageous over the existing single-production processes.

Finally, we compared the utility loads for the single-production and co-production processes, as shown in Table 6. The DMM single-production process has minimal utility load because there is no separation of azeotropes. Single-production DMC has the largest utility load due to problems with azeotrope separation and low methanol conversion. By introducing the process of DMM and DME generation, the utility load of the 3DM co-production process is significantly reduced relative to the DMC single-production process.

## 6. Conclusions

In DMC production, DMC and methanol form an azeotrope, and the separation of them consumes a large amount of energy. In this paper, the combination of DMC production with DMM and DME productions was proposed. The resulting 3DM co-production process was investigated using the Aspen Plus models and compared with the DMC, DMM, and DME single-production processes. Under optimal conditions, the 3DM co-production process can produce DMC, DME, and DMM with a mass concentration of 99.9%. The exergy analysis was carried out on the 3DM co-production process and compared with the DMC, DME, and DMM single-production processes. The results indicate that the 3DM co-production process has noticeable advantages in internal exergy destruction in comparison with the single-production processes and can greatly reduce internal exergy destruction. By combining the DMC production with DMM and DME productions, the subsequent separations are more energy efficient, and the internal exergy destruction is significantly reduced. To produce a product of the same composition, the exergy destruction using the 3DM co-production process is about 276% less than those using the DMC, DMM, and DME single-production processes. The exergy efficiencies ${\;\varphi }_{}\;$ of the 3DM co-production process are greatly improved compared with the DMC single-production process. The utility loads of the co-production process are significantly lower than that of the single-production process. The methanol conversion ratio of the 3DM co-production process can reach 95%, which is significantly higher than the single-production process. The developed 3DM production produces DMC, DMM, and DME, and overcomes the disadvantages of the existing DMC production method and possesses attractive energy efficiency. The strategy of “reaction instead of separation” is feasible.

## Figures and Tables

**Table 1:** Catalysts and their performances in oxidative carbonylation of methanol to DMC.

Catalysts	C_MeOH_ (%)	S_DMC_ (%)	${\mathrm{STY}}_{\mathrm{DMC}}(mg\;\cdot \;g_{cat}^{-1}\;\cdot \;h^{-1})$	References
PdCl_2_-CuCl_2_-KOAc/AC	12%	80%	747	[5]
Cu/AC	1.8%	73%	150	[6]
CuY zeolite	11.4%	23.7%	-	[7]
CuCe/AC	4.1%	85.2%	143.3	[8]
CuY	18.9%	50%	525.1	[9]
Cu_2_(OH)_3_Cl/AC	6.9%	67.3%	139.1	[10]

**Table 2:** The standard chemical exergy of the components.

Component	The Standard Molar Chemistry Exergy (kJ/mol)
CH_3_OH	716.192
DME	1415.126
CO	275.323
O_2_	3.955
H_2_O (g)	8.580
H_2_O (L)	0.000
DMC	1490.526
DMM	1979.799

**Table 3:** The energy and exergy balance equations for the units in the 3DM co-production.

The Operating Unit	Energy Balance Equation	Exergy Balance Equation
The RStoic module	$\sum {\dot{m}}_{in}h+Q_{r}=\sum {\dot{m}}_{out}h+Q_{c}$	$\sum B_{in}+B_{u}{}_{}^{}=\sum B_{out}+B_{d}$
The Sep module	$\sum {\dot{m}}_{in}h=\sum {\dot{m}}_{out}h$	$\sum B_{in}=\sum B_{out}+B_{d}$
The RadFrac module	$\sum {\dot{m}}_{in}h+Q_{r}=\sum {\dot{m}}_{out}h+Q_{c}$	$\sum B_{in}+B_{u}=\sum Ex_{out}+B_{d}{}^{}$
The Heater module	$\sum {\dot{m}}_{in}h+Q_{r}=\sum {\dot{m}}_{out}h+Q_{c}$	$\sum B_{in}+B_{u}=\sum B_{out}+B_{d}$
The HeatX module	$\sum {\dot{m}}_{in}h=\sum {\dot{m}}_{out}h$	$\sum B_{in}=\sum B_{out}+B_{d}$
The Mixer module	$\sum {\dot{m}}_{in}h=\sum {\dot{m}}_{out}h$	$\sum B_{in}=\sum B_{out}+B_{d}$
The Pump module	$\sum {\dot{m}}_{in}h+W=\sum {\dot{m}}_{out}h$	$\sum B_{in}+B_{w}=\sum B_{out}+B_{d}$

**Table 4:** The internal exergy destruction the per unit products in the proposed 3DM process in comparison with the single-production processes.

Process	The DMC Single-Production Process	The DMM Single-Production Process	The DME Single-Production Process	The 3DM Co-production Process
Exergy destruction (kJ/mol)	2477.433	1977.543	75.255	324.143

**Table 5:** Exergy efficiencies for DMC, DMM, and DME productions.

Process	The 3DM Co-Production	The DMC Single-Production	The DMM Single-Production	The DME Single-Production
Exergy efficiencies φ	78.92%	37.56	85.88%	94.76%

**Table 6:** Utility loads for DMC, DMM and DME productions.

Process	The 3DM Co-Production	The DMC Single-Production	The DMM Single-Production	The DME Single-Production
Loads of Hot utility (kJ/mol *)	382.4205	6818.432	134.4326	473.578
Loads of Cold utility (kJ/mol *)	506.4113	7356.62	132.4123	709.0614
Electricity consumption (kJ/mol *)	0.41458	222.4987	2.370107	0.743755

* The used loads to produce each mol of product. The product of The 3DM co-production is the sum of the three products.

## Data Availability

Not applicable.

## Supplementary Materials

The following supporting information can be downloaded at: https://www.mdpi.com/article/10.3390/e24101438/s1, Table S1: Material balance calculation in the 3DM co-production process; Table S2: Exergy of the streams in the 3DM co-production process; Table S3: Material balance calculation in the DMC single-production process; Table S4: Material balance calculation in the DMM single-production process; Table S5: Material balance calculation in the DME single-production process; Table S6: Introduction to used the modules of Aspen Plus.

## Nomenclature

B	exergy rate of stream, kW
N	molar flow rate, mol/s
ex	specific molar exergy, kJ/mol
$C_{MeOH}$	conversion ratio of methanol
$S_{DMC}$	selectivity of DMC
$STY_{DMC}$	space-time yield of DMC, $mg\cdot g_{cat}^{-1}\cdot h^{-1}$
H	enthalpy, kJ/mol
S	entropy, kJ/(mol·K)
$y_{i}$	mole fraction of component i
$\dot{Ex}$	standard chemical exergy, mol/s
T	temperature, K
R	universal gas constant, kJ/(mol·K)
$∆G_{f}$	Gibbs free energy variation in the direction of a given chemical Reaction, kJ/mol
n	number of moles of element
$\dot{m}$	mass flow rate, kg/s
h	specific enthalpy, kJ/kg
$\varphi_{1}$	exergy efficiencies
$\dot{Q}$	heat transfer rate, kW
$\dot{W}$	work rate, kW
$∆H_{R}$	Reaction Heat, kJ/mol
0	ambient reference
ch	chemical
ph	physical
ki	kinetic
po	potential
in	feed stream
out	outbound stream
d	destruction
el	element
r	high-grade heat sources
c	low-grade heat sources
p	products
u	utilities
