# Supplementary material for: Co-Production of Dimethyl Carbonate, Dimethoxymethane and Dimethyl Ether from Methanol: Process Design and Exergy Analysis
Source: Entropy (Basel). 2022 Oct 9;24(10):1438. doi: 10.3390/e24101438 (PMC9601992; doi:10.3390/e24101438)
Supplement: Supplementary file 1 [file entropy-24-01438-s001.zip › entropy-1900181-supplementary.pdf]

entropy MDPI

List of supporting information

Table S1. Material balance calculation in the 3DM co-production process.
Table S2. Exergy of the streams in the 3DM co-production process.
Table S3. Material balance calculation in the DMC single-production process.
Table S4. Material balance calculation in the DMM single-production process.
Table S5. Material balance calculation in the DME single-production process.
Table S6. Introduction to used the modules of Aspen Plus.

Table S1. Material balance calculation in the 3DM co-production process.

Streams Name	Temperature (℃)	Pressure (MPa)	Mass Flow （kg/hr）	Mass Fraction						
				CH_3OH	DMC	DME	DMM	CO	O_2	H_2O
**DMC**	**25.00**	**0.10**	37.500	0.000	1.000	0.000	0.000	0.000	0.000	0.000
DME	-25.05	0.10	65.175	0.000	0.000	1.000	0.000	0.000	0.000	0.000
DMM	25.00	0.10	109.997	0.000	0.001	0.000	0.999	0.000	0.000	0.000
FEED	25.00	0.10	344.357	0.744	0.000	0.000	0.000	0.163	0.093	0.000
G-BLEED	40.00	0.10	46.514	0.000	0.000	0.000	0.000	0.953	0.047	0.000
H_2O	25.00	0.12	85.158	0.000	0.001	0.000	0.000	0.000	0.000	0.999
RE-CH_3OH	67.15	0.12	11.789	0.977	0.000	0.000	0.023	0.000	0.000	0.000
S1	14.27	0.10	542.203	0.494	0.000	0.000	0.001	0.430	0.075	0.000
S2	50.37	0.10	542.203	0.494	0.000	0.000	0.001	0.430	0.075	0.000
S3	83.04	0.10	542.203	0.494	0.000	0.000	0.001	0.430	0.075	0.000
S4	130.00	0.10	542.203	0.494	0.000	0.000	0.001	0.430	0.075	0.000
S5	160.00	0.30	542.200	0.445	0.069	0.000	0.001	0.409	0.063	0.014
S6	130.00	0.30	542.200	0.445	0.069	0.000	0.001	0.409	0.063	0.014
S7	130.00	0.10	542.200	0.445	0.069	0.000	0.001	0.409	0.063	0.014
S8	129.85	0.10	542.200	0.189	0.069	0.000	0.203	0.409	0.020	0.110
S9	220.00	0.10	542.200	0.189	0.069	0.000	0.203	0.409	0.020	0.110
S10	220.00	0.10	542.197	0.021	0.069	0.120	0.203	0.409	0.020	0.157
S11	47.24	0.10	542.197	0.021	0.069	0.120	0.203	0.409	0.020	0.157
S12	40.00	0.10	542.197	0.021	0.069	0.120	0.203	0.409	0.020	0.157
S13	40.00	0.10	232.571	0.000	0.000	0.000	0.000	0.953	0.047	0.000
S14	40.00	0.10	186.057	0.000	0.000	0.000	0.000	0.953	0.047	0.000
S15	40.00	0.10	309.631	0.037	0.122	0.210	0.356	0.000	0.000	0.275
S16	72.54	0.10	244.456	0.047	0.154	0.000	0.451	0.000	0.000	0.348
S17	72.56	0.12	244.456	0.047	0.154	0.000	0.451	0.000	0.000	0.348
S18	41.05	0.10	121.797	0.095	0.001	0.000	0.905	0.000	0.000	0.000
S19	30.99	0.10	197.950	0.079	0.000	0.038	0.883	0.000	0.000	0.000
S20	30.59	0.10	197.950	0.079	0.000	0.038	0.883	0.000	0.000	0.000
S21	32.38	1.10	197.950	0.079	0.000	0.038	0.883	0.000	0.000	0.000
S22	104.42	1.00	87.953	0.178	0.000	0.084	0.737	0.000	0.000	0.000
S23	18.23	0.10	76.164	0.055	0.000	0.098	0.848	0.000	0.000	0.000
S24	131.59	1.10	109.997	0.000	0.001	0.000	0.999	0.000	0.000	0.000
S25	25.00	1.10	109.997	0.000	0.001	0.000	0.999	0.000	0.000	0.000
S26	94.91	0.10	122.659	0.000	0.306	0.000	0.000	0.000	0.000	0.693
S27	94.96	0.15	122.659	0.000	0.306	0.000	0.000	0.000	0.000	0.693
S28	101.64	0.15	228.389	0.000	0.492	0.000	0.092	0.000	0.000	0.417
S29	78.54	0.10	143.231	0.000	0.783	0.000	0.146	0.000	0.000	0.071
S30	80.09	1.10	143.231	0.000	0.783	0.000	0.146	0.000	0.000	0.071

S31	166.60	1.00	105.730	0.000	0.707	0.000	0.198	0.000	0.000	0.096
S32	104.82	0.12	85.158	0.000	0.001	0.000	0.000	0.000	0.000	0.999
S33	187.30	1.08	37.500	0.000	1.000	0.000	0.000	0.000	0.000	0.000
S34	25.00	1.08	37.500	0.000	1.000	0.000	0.000	0.000	0.000	0.000

Table S2. Exergy of the streams in the 3DM co-production process.

Streams Name	Molar Flow（kmol/h）	Physical Exergy (kW)	Chemical Exergy (kW)	Total Exergy (kW)
S-1	17.966	2292.320	0.125	2292.445
S-2	17.966	2292.320	3.124	2295.444
S-3	17.966	2292.320	3.871	2296.191
S-4	17.966	2292.320	5.829	2298.149
S-5	17.339	2266.253	21.006	2287.259
S-6	17.339	2266.253	19.252	2285.505
S-7	17.339	2266.253	6.135	2272.389
S-8	16.617	2161.620	8.211	2169.830
S-9	16.617	2161.620	14.706	2176.325
S-10	16.617	2158.057	15.503	2173.560
S-11	16.617	2146.804	0.935	2147.738
S-12	16.617	2146.804	0.388	2147.192
S-13	8.254	198.942	0.000	616.230
S-14	6.603	483.498	0.020	483.517
S-15	8.363	1550.202	0.289	1550.491
S-16	6.948	996.659	0.842	997.502
S-17	6.948	996.659	0.843	997.502
S-18	1.809	827.458	0.059	827.517
S-19	2.949	1359.007	0.027	1359.035
S-20	2.949	1359.007	0.022	1359.030
S-21	2.949	1359.007	0.032	1359.040
S-22	1.503	604.756	0.875	605.631
S-23	1.140	531.749	0.007	531.756
S-24	1.446	754.602	1.503	756.105
S-25	1.446	754.602	0.000	754.602
S-26	5.139	171.880	0.956	172.836
S-27	5.139	171.880	0.957	172.837
S-28	6.805	656.763	2.622	659.385
S-29	2.082	657.968	0.423	658.391
S-30	2.082	657.968	0.448	658.415
S-31	1.665	485.692	2.282	487.974
S-32	4.723	0.408	1.010	1.418
S-33	0.416	172.364	0.833	173.197
S-34	0.416	172.364	0.000	172.364
RE-CH_3OH	0.363	73.360	0.049	73.409
DMC	0.416	172.364	0.000	172.364
DME	1.415	556.102	0.116	556.218
DMM	1.446	754.602	0.000	754.602
FEED	11.000	1739.965	0.033	1739.997
G-BLEED	1.651	120.874	0.005	120.879
H_2O	4.723	0.408	0.000	0.408

Table S3. Material balance calculation in the DMC single-production process.

Streams Name	Temperature (℃)	Pressure (MPa)	Mass Flow (kg/hr)	Mass Fraction CO	CH_3OH	DMC	H_2O	O_2
**S-1**	**17.21**	**0.10**	71513.38	0.337	0.448	0.022	0.000	0.193
S-2	66.85	0.10	71513.38	0.337	0.448	0.022	0.000	0.193
S-3	187.12	0.30	71513.49	0.337	0.448	0.022	0.000	0.193
S-4	50.35	0.30	71513.49	0.318	0.403	0.085	0.013	0.181
S-5	34.85	0.30	71513.49	0.318	0.403	0.085	0.013	0.181
S-6	34.85	0.30	35826.01	0.000	0.805	0.170	0.025	0.000
S-7	63.40	0.10	34925.20	0.000	0.826	0.174	0.000	0.000
S-8	63.42	0.12	34925.20	0.000	0.826	0.174	0.000	0.000
S-9	68.38	0.12	49286.98	0.000	0.872	0.128	0.000	0.000
S-10	63.26	0.10	18866.39	0.000	0.750	0.250	0.000	0.000
S-11	63.93	1.10	18866.39	0.000	0.750	0.250	0.000	0.000
S-12	128.38	0.80	14361.78	0.000	0.986	0.014	0.000	0.000
S-13	30.00	0.84	4504.61	0.000	0.000	1.000	0.000	0.000
S-14	174.34	0.84	4504.61	0.000	0.000	1.000	0.000	0.000
S-15	160.00	0.30	71513.49	0.318	0.403	0.085	0.013	0.181
S-16	104.83	0.12	900.81	0.000	0.000	0.000	1.000	0.000
DMC	30.00	0.10	4504.61	0.000	0.000	1.000	0.000	0.000
FEED	25.00	0.10	5405.31	0.259	0.593	0.000	0.000	0.148
H_2O	30.00	0.12	900.81	0.000	0.000	0.000	1.000	0.000
RE-CH_3OH	68.65	0.12	30420.59	0.000	0.948	0.052	0.000	0.000
RE-G	34.85	0.30	35687.48	0.636	0.000	0.000	0.000	0.364

Table S4. Material balance calculation in the DMM single-production process.

Streams Name	Temperature (℃)	Pressure (Mpa)	Mass Flow (kg/hr)	Mass Fraction CH_3OH	O_2	DMM	H_2O
**S-1**	**32.13**	**0.08**	18613.59	0.894	0.091	0.000	0.015
S-2	119.85	0.08	18613.59	0.894	0.091	0.000	0.015
S-3	180.00	0.10	18613.59	0.379	0.005	0.408	0.208
S-4	61.61	0.10	18613.59	0.379	0.005	0.408	0.208
S-5	10.00	0.10	18613.59	0.379	0.005	0.408	0.208
S-6	10.00	0.10	18524.55	0.381	0.000	0.410	0.209
S-7	10.00	0.10	84.60	0.000	1.000	0.000	0.000
S-8	46.42	0.10	14927.90	0.472	0.000	0.508	0.019
S-9	99.61	0.10	3596.65	0.001	0.000	0.000	0.999
S-10	40.77	0.08	16652.83	0.454	0.000	0.529	0.017
S-11	35.04	0.08	9336.42	0.056	0.000	0.944	0.000
S-12	38.65	3.00	9336.42	0.056	0.000	0.944	0.000
S-13	169.31	2.80	1740.60	0.299	0.000	0.701	0.000
S-14	169.31	2.80	1725.16	0.292	0.000	0.708	0.000
S-15	169.31	2.80	15.44	1.000	0.000	0.000	0.000
S-16	184.27	2.80	7595.82	0.001	0.000	0.999	0.000
S-17	30.00	2.80	7595.82	0.001	0.000	0.999	0.000
DMM	30.00	0.10	7595.82	0.001	0.000	0.999	0.000
FEED	25.00	0.10	11212.59	0.857	0.143	0.000	0.000
G	10.00	0.10	4.45	0.000	1.000	0.000	0.000

H_2O	35.00	0.10	3596.65	0.001	0.000	0.000	0.999
RE-CH_3OH	59.95	0.08	7316.41	0.961	0.000	0.000	0.039
RE-O_2	10.00	0.10	89.04	0.000	1.000	0.000	0.000

Table S5. Material balance calculation in the DME single-production process.

Streams Name	Temperature (℃)	Pressure (Mpa)	Mass Flow (kg/hr)	Mass Fraction		
				CH_3OH	DME	H_2O
**S-1**	**200.00**	**0.10**	3620.91	0.998	0.000	0.002
S-2	220.00	0.10	3620.91	0.113	0.636	0.251
S-3	50.00	0.12	3620.91	0.113	0.636	0.251
S-4	86.42	0.12	1317.47	0.310	0.001	0.690
S-5	236.50	0.12	3620.91	0.113	0.636	0.251
S-6	63.00	0.10	3620.91	0.998	0.000	0.002
S-7	183.82	0.12	3620.91	0.113	0.636	0.251
S-8	104.82	0.12	900.78	0.000	0.000	1.000
S-9	29.74	0.10	3620.91	0.998	0.000	0.002
DME	-24.93	0.10	2303.44	0.000	1.000	0.000
FEED	25.00	0.10	3204.22	1.000	0.000	0.000
H_2O	35.00	0.12	900.78	0.000	0.000	1.000
RE-CH_3OH	62.56	0.10	416.69	0.979	0.002	0.020

Table S6. Introduction to used the modules of Aspen Plus.

Module	Explanation	Function	Applicable Object
Rstoic	Stoichiometric reactor	Reactor module simulating known reaction degree and conversion rate	Reactors with unknown or unimportant reaction kinetic data but known stoichiometric coefficients of chemical reactions and degree of reaction
Sep	component splitter	To divide the inlet logistics into multiple outlet logistics according to the specified component flow or fraction	Component separation operation, but detailed separation process is unknown or unimportant when
RadFrac	Strict calculation module of single tower distillation	Strict check and design calculation of a single distillation column	Conventional distillation, absorption, stripping, extractive distillation, azeotropic distillation, three-phase distillation, reactive distillation, etc.
Heater	Heater or Cooler	Determination of Thermodynamic State and Phase State of Export Logistics	heater, cooler, condenser, etc
HeatX	Two-stream logistics heat exchanger	Simulate heat transfer between two streams of logistics	Two-stream heat exchanger, check the known structure of the shell and tube heat exchanger, using strict program simulation of the shell and tube heat exchanger, air cooler and plate heat exchanger
